# State Medicine in Great Britain
*Essays on State Medicine. By H. W. Rumsey. London: 1856. Churchhill.Rapports Généraux des Travaux du Conseil de Salubrité pendant les années 1846 à 1848. Publiés par ordre de M. le Préfet de Police. Paris: 1855. Over Legislation. The Westminster Review, July 1852.


**Published:** 1856-07

**Authors:** 


### JOURNAL OF PUBLIC HEALTH.

JULY 1856.

### STATE MEDICINE IN GREAT BRITAIN.*

The past quarter, from the number of works that have been added to sanitary literature, has been one of peculiar interest to the advocate of sanitary science. Foremost amongst these works is the volume of Mr. Rumsey on *State Medicine.* Until the appearance of this important volume, we had in the English tongue no comprehensive treatise on the all-engrossing topic discussed in its pages; and we are bound to say, however much we are inclined to dispute the views of its author, that for evidence of careful research, unwearied industry, calm judgment, manly expression of opinion, and originality of thought, the book is unexceptionable.

Bringing into free play a thorough knowledge of the laws of life, health, and disease, an acquaintance with all that has been done to advance sanitary measures in the united kingdom, and the energies of an enthusiastic mind, Mr. Rumsey has approached his novel task with the firmness of one who writes only on subjects which he has long and perseveringly studied; and as the reader of the *Essays* becomes more and more impressed with this fact as each page is perused, he sinks for the moment all differences of feeling, that he may follow the writer to the end of his argument. This point reached, the war of opinion begins; and if we in any degree understand the sentiments of the reading and thinking community, we fear our author will have to concede many modifications before he concludes a peace with the disciplined enemies who wait for him.

The work itself is made up of six essays. The first is entitled "The Outline of a Sanitary Code"; the second, "Edu-

---

* Essays on State Medicine. By H. W. Rumsey. London: 1856. Churchhill.

Rapports Généraux des Travaux du Conseil de Salubrité pendant les années 1846 à 1848. Publiés par ordre de M. le Préfet de Police. Paris: 1855.

Over Legislation. The Westminster Review, July 1852.

cation in the Healing and Health-preserving Arts"; the third, "Sanitary Inquiry"; the fourth, "Medical Care of the Poor"; the fifth, "Local Sanitary Legislation"; and the sixth, "Health Police in relation to Local Sanitary Administration."

The first of these essays contains the substance of Mr. Rumsey's political plans and suggestions. With great labour and precision he lays down an outline of a sanitary code. It would be vain to attempt here to follow him in all his details; but reviewing the essay as a whole, its teaching is, that the English people should be made to accept the practice of sanitarian principles, under the influence of the control and direction of the government; that the government should organize one scientific central sanitary board, and other local boards in various districts of the country; that these boards, central and local, should have the care of the public health; that they should have power to act, to do, and to perform whatever they in their sanitary wisdom may consider necessary for the physical welfare of the subject; and that the said subject should be bound to submit, whether he understandeth or not, because it is all for his good, as he will see if he will wait and build largely on simple faith.

This is the key of our author's position, his Malakoff in true Russian sense. It is the proposed application to sanitary improvement of an old principle—govern, govern, govern. If it is necessary for a brother's health, as one shall think, that he rest his head for a few hours on a charmed pillow, go not by the roundabout way of proving to your brother how excellent is the plan you name, but down with him by your muscular arm, and if he rebel, enact Mrs. Jael, and put a nail through his temples to pin him safely to his good. Or, if a life-exhausted man shall solemnly say "he cannot swallow, he would rather die quietly", let him not die quietly, but fill him with wine; for is it not falser charity to leave him alone, than to choke him in administering the supposed—supposed only —*proper* restorative? In a word, strong men all, be strong and firm and resolutely active in driving your fellow men out of pigdom on to the delectable mountains. We say that Mr. Rumsey's plan is neither special nor novel; it is founded on the popular conviction that governments not only ought, but that they can do and compass all things. Yet what a radical error is here committed, let all who will observe say. The common fault-cry against the government is that it will not shake off routine, that it will not advance. Deputations press on ministers great public projects and improvements, and ministers receive deputations with smiles and courtesies, and ministers approve of the spirit of the projects, and will consider them. Speculative geniuses in science and in art press on government boards and officials various suggestions, inventions, and plans; government boards and officials are obliged to the speculative geniuses for such suggestions, and will consider them or refer them. But neither the suggestion of the deputation, nor the work of science or art, gets much in the end from the government, although the government in its turn gets many hard blows for its negligence. These blows are, however, serenely borne, for they fall on innocent shoulders; it is not negligence, or indolence, or want of conscience, or lack of desire to do good, that holds the ministerial mind in check, but the oppression of over work, and the sensation of having tasks already before it, which it has not the time or the ability to perform. It is with governments as with individuals; the nonrealisation of labours which may be attempted, but which are by their vastness impossible, is too often accepted as a sign of innate weakness or of culpable neglect.

Again, in England, the administrative labours of our government are not only in themselves overpowering, but are by the nature of our constitution often entrusted to an ability of a second or even a third rate order. In our band of rulers there may be one or two men of great intellect and vigour, but that the majority of the band should perform their duties in such a way as to prove themselves more competent than the majority of the educated lookers-on amongst the masses, is impossible. And as it is impossible, so is it wise; for such is the force and the fascination of great mental talent, and such the extent of its own self-aggrandisement, that once placed in the position to know no hindrance, its ambition is unrestrained and its usurpation inevitable. Conscious of their own inherent strength, and of their power to guide their own actions, to support their own growth, to cultivate their own minds, the British people need dread no event so much as that of seeing brilliant ambition and overpowering intellectual force at work in the state. Liberal men with good common sense, armed with a desire to follow the instincts, the industry, and the learning of the people, and jealous ever for the honour and safety of their country, are here the only legislators required or desirable; and if it is to be lamented, on the one hand, that we do not always obtain even these qualities in our rulers, it is a source of satisfaction, on the other, to know that the *summum bonum* of legislative skill is in such qualities embodied.

That the legislature of Great Britain does not recognise the simplicity and unity of its true functions, is no argument against the correctness of the views we have propounded. On the contrary, it is seen and tolerably well understood, that whenever the government steps out of its simple administrative powers, in relation to war, peace, or those other affairs in which the mere administration of the national will is concerned, to legislate on our domestic or minor social concerns, it is plunged into difficulties which it cannot surmount and had better have left alone. The result is, if anything in such cases is done at all, that what is done is a compromise, or "a dab of legislation", which creates a furor for a moment, and then sinks down into a failing impracticable scheme, requiring a new "dab" every year, in the shape of an amendment, to keep it from sheer dissolution.

Amongst those who will unite with us, in thus criticising the evils of puny legislation, none will join more heartily than the author of the *Essays on State Medicine.* He acknowledges that "dabs" of legislation are beneath contempt, in all that concerns the preservation of the public health; and his *Essays* are themselves written mainly to show what a comprehensive measure of sanitary reform is open for construction, what a State Medicine there might be in England, if an English government would be bold enough to grapple with the whole question, draw up their own terms, and make the nation sign them. It cannot but be interesting to follow our author at this point through some of his suggestions, and see what the State, not every man individually, should do for the health of Fatherland.

First, the State should direct certain investigations bearing on public health, and relating to statistical, topographical, and jurisprudential questions; it should institute practical arrangements for personal health; and it should organise a machinery for carrying into effect the aforesaid investigations, and for the administration of the laws which might come under the State Medicine system.

In enumerating the details of his project, it is but fair to Mr. Rumsey to say, that he makes no one suggestion that would not be beneficial to the community if properly and spontaneously carried out. He has the clearest ideas as to the essentials for healthy and comfortable homes. He proposes model advantages for his race: fresh air, green trees, the cleanly threshold, the pure drinking stream, the unpoisoned food, the healthy marriage union, the innocent and vigorous exercise of the mind and body, recreation and relaxation from toil, the emancipation of wretched women from the abyss of pollution, the due disposal of the dead, the possession of hospitals and almshouses everywhere for the sick and the destitute, the subjection of all classes of the people to scientific medical supervision, and the suppression of the leading causes of disease both in man and in his lower earthmates.

How fully we coincide with Mr. Rumsey as to the advantages that would follow from the development of these suggestions, we need not say. But we look upon him as one who, seeing the goal, is so unfortunate in groping for the road that leads to it, as to walk continually around it, ever in the circle. He sees what is excellent to man, yet in so seeing he ignores man himself, his individuality, his tastes, his education, his rights. He assumes that by beginning to cast into perfect shape the dead matter, he can end by conquering the living mind. He erects the model dwelling-place, not for the man and his gratefully acknowledged happiness, but for the State, with its paternal and rigidly enforced system.

On the subject of "Education in the Healing and Health Preserving Arts", the author of the *State Medicine* is so broad and sound in some of his views, that the most philosophic liberal might agree with him in many essential points. But here, again, the idols of governmental power, wisdom, and authority, bewitch and embarrass the writer. Obviously assuming as a basic fact that which is no fact at all, namely, that governments are composed of the wisest, the most enlightened, the most unselfish members of the community, we are again goaded with the idea that the State must superintend every matter connected with medical education. Wanted immediately, a Medical Circumlocution Office, with an inferior descendant of the Tite Barnacles for the president, a young Barnacle for the secretary, a nephew Barnacle for the clerk, and a medical council composed of the highest talent, for performing the important duties of dummies. But, on hypothetical grounds (for practical men would not entertain the question), what should the Medical Circumlocution Office do? First it should give its license to practise to all whose preliminary general and medical education is proved to be sound, and whose moral character and habits are in keeping with the requirements of the profession. This, as regards protection of medical men, is the extent to which protection should be carried. At the same time the State, or rather the office aforesaid, should train and educate youths for medical and sanitary employments, "regulate the number of admissions into the profession by the demand for medical employment", and signify the age at which our aspirants for medical practice should commence their practical career.

As regards the right of the State to interfere in these matters, our author advances one "conclusive and only unanswerable plea", namely, that THE SAFETY OF THE PEOPLE IS THE SUPREME LAW OF THE LAND. We grant this position; but who are to be the law-givers? to whom is the health of the people to be entrusted? to the people themselves, or to a small section of the people, in intellect not a hair's breadth above the educated community? Shall the will of this minority, simply because its objects are good, be absolute, though it kick at common sense, put fetters on liberty, and ignore those grand educational cravings by satisfying which men progress in peace?

The great evil of medicine now is, that she is over-legislated for; that she has more masters than she can obey; that some score or so of separate boards have not only the right to grant certificates for some form of practice or title, but have certain restrictive privileges, which they know well how to use. So far from more restrictions of this kind being imposed on medicine, she requires to be freely relieved of such heavy burdens, and to be permitted to stand in her own strength, untrammeled by supposed supports. Permit this freedom, and she is safe enough; but place her on the crutches of the State, let her never feel her own weight, or enjoy an unrestrained exercise, and, her limbs withering, she must become a hopeless State cripple, with a good clear head, of great use to mankind in general in a scientific way, but, as a political fact, a weak, hobbling, top-heavy body, to be looked down at by the parliamentarian, to be sneered at by the wig and gownsmen, to be laughed at by the mob.

We do not deny, however, that in the present confused state of medical affairs, order is required; and that a few simple and uniform rules, regulating the studies of students and of examination, are necessary. But from the profession itself these reforms must come; they must be shaped in accordance with the liberal and progressive spirit of the time; and they must in themselves proclaim silently, but convincingly, that there is in medicine a truth which, shunning no inquiry, and fearing no rivalry, proves its own worth.

On Sir Benjamin Hall's new Act for the better Local Management of the Metropolis, Mr. Rumsey hits mercilessly hard. Why are, he inquires, in this Act, the existing corps of sanitary officers, the medical attendants of the poor, left unrecognised? As doctors differ, are these functionaries to get up lively discussions with the new officers of health on those controverted points of management which continually arise in a locality? Why have local boards, which know nothing about sanitary duties, the disposal of sanitary offices? Can the officer of a local board be independent? Will not the new plan give rise in the end to a modified "Tender System"? These are fair and reasonable questions; but, under any system whatever, would not other questions, quite as difficult to meet, spring up? It is not in the capacity of any two, or any fifty men to draw up a code for the guidance of other men, that shall be free from objections more or less heavy, or from blundering more or less foolish. Whatever act or deed is from man, is of man, takes from his mind its form, and is in itself an independency. But appeals it not also to minds equally independent, and of the which no two (so Nature rules) ever absolutely agree?

And here, indeed, is the difficulty and the perversity of most political suggestions—saving those that harmonise, educate, and urge, in the gentle and firm voice of humanity, the principles of self-trust and of confidence in nature—here is the difficulty, that the mind of man is an isolation; that no two men can agree; that no two epochs of men can be found which have agreed; that, to attain the same object, each man takes a different, an individual course.

For England an arbitrary system of State Medicine is impossible; there are not the legislators to conceive it or direct it. Grant as a fact the formation of such a system, in a comprehensive form, as its advocates say, and an *imperium in imperio* would be the upshot,—the physician-administrators of which might soon look out for the fate of their Roman prototypes—an universal dislike, and an expulsion from the community.

But what if an arbitrary system of State Medicine were consistent with the spirit and temper of the English people? Would it then do good, or advance the position of the nation? Impossible. An ignorant mass can never be governed into knowledge; a rigidly governed mass can never exist long, unless it be and continue ignorant. The science of politics, like that of chemistry, has its elements, which have their repulsions, their affinities, and their combinations. Concerning some of these it may be said that, while the union of ignorance and obedience is often slight, and the union of knowledge and rigid obedience is impossible, the union of independence and of knowledge is not only the firmest of all political combinations, but supplies a radical principle, elementary in its characters, which neither the furnace of the bigot nor the hammer of the despot can separate or destroy.

In England we want no State Medicine. The very word is deadening in its import. If medicine, preventive and curative, cannot live as an educational fact, and as a simple guide to health, to wealth, to happiness; if she cannot appeal to the intellect, the reason, the senses, the conscience, then must medicine die; for, in the absence of these vital forces, no decoction of parchment, dispensed from St. Stephen's, can save her. The individual man, armed with the knowledge and with the genius for demonstration, requires no Act of Parliament to enforce his claims on the world. In the broad light of day, he lays his thoughts and his proofs before his fellow-men; and, if the proofs are convincing, and the thoughts good, they make their way, they stand alone; and, if they are of the best, they stand in the end where the best should—*first.* The same rule applies to men in their collective relations; the strength, the influence, of the mass is given in the unit.

In the other sciences this rule also holds supremacy. What would be thought of a State astronomy, or a State chemistry? The idea is monstrous. These sciences live by their own wits; why, then, should not medicine?

But can the State do nothing for medical science? It can; not, it is true, in the way of prompting it with anathemas and so-called rights, not by hugging it in its arms like an unhealthy infant, but by treating it as the representative of a vigorous manhood, requiring but encouragement to be able to sustain a high and useful position in the world. The State could mete out rewards and honours to discoveries in medical science; it could give to the distinguished in medicine a seat in its councils, and be all the healthier itself for the infusion; it could lend to medicine its great assistance in the collection of facts; it could increase the importance of the Registrar-General's Reports, by adding to the duties of that important functionary the additional duty of collecting statistics of disease; it could establish meteorological stations in various parts of the country; it could at times peremptorily remove any known and removable cause of disease; it could abolish one of its own glaring iniquities—the endorsement of the quack's poison with Her Majesty's seal; it could with advantage institute a law and medicine tribunal or board of reference, for its own guidance, in cases where life or health is concerned; and on the ministers of that tribunal it could confer its best honours.

These suggested modifications may not include all that are necessary, but they have the advantage of being all practical; and if they were fairly and simply explained and asked for, they could be obtained in time. But so long as men living in a world of their own go to our legislators, as if the legislators could themselves do what the nation will not hear of, and ask for the institution of a new power in the State, so long is it to be expected that what is called State Medicine will remain, in so far as Great Britain is concerned, a mere verbal curiosity, full of sound and energy, signifying nothing.

---

